# Scedosporiosis in a Combined Kidney and Liver Transplant Recipient: A Case Report of Possible Transmission from a Near-Drowning Donor

**DOI:** 10.1155/2016/1879529

**Published:** 2016-12-13

**Authors:** Rachael Leek, Erika Aldag, Iram Nadeem, Vikraman Gunabushanam, Ajay Sahajpal, David J. Kramer, Thomas J. Walsh

**Affiliations:** ^1^Department of Abdominal Transplant, Aurora St. Luke's Medical Center, Milwaukee, WI, USA; ^2^University of Wisconsin School of Medicine and Public Health, Madison, WI 53726, USA; ^3^Department of Critical Care, Aurora St. Luke's Medical Center, Milwaukee, WI, USA; ^4^Weill Cornell Medicine, Cornell University and New York Presbyterian Hospital, New York, NY, USA

## Abstract

*Scedosporium *spp. are saprobic fungi that cause serious infections in immunocompromised hosts and in near-drowning victims. Solid organ transplant recipients are at increased risk of scedosporiosis as they require aggressive immunosuppression to prevent allograft rejection. We present a case of disseminated* Scedosporium apiospermum* infection occurring in the recipient of a combined kidney and liver transplantation whose organs were donated by a near-drowning victim and review the literature of scedosporiosis in solid organ transplantation.

## 1. Introduction

*Scedosporium *is a saprobic fungus that naturally occurs in soil, manure, sewage, and water bodies polluted by environmental contaminants. Cases of infections with* Scedosporium *spp. are reported worldwide and can range in severity from colonization or local infection to disseminated disease [1–5].* S. apiospermum* most commonly infects the lungs as inhalation is often the suspected mode of organism acquisition [6].

The genus* Scedosporium* includes, but is not limited to, three species that cause life-threatening infections in humans:* Scedosporium apiospermum*,* Scedosporium prolificans *(recently renamed* Lomentospora prolificans*), and* Scedosporium aurantiacum *[7].* Scedosporium *spp. appear as branching septate hyphae when isolated on standard culture media.

*Scedosporium *spp. have been implicated in near-drowning accidents [8–10]. Indeed* Scedosporium *spp. are the most common cause of fungal pneumonia, infection of the central nervous system, and disseminated disease following near-drowning events. There are few cases of donor to recipient transmission of infection of* Scedosporium *spp. and even fewer reports of near-drowning donor transmission of diseases [11, 12]. We report the clinical course and management of disseminated* S. apiospermum* infection in a combined kidney and liver transplantation case receiving organs from a brain-dead donor who suffered a near-drowning accident; we further review the literature on scedosporiosis in solid organ transplant recipients.

## 2. Case Presentation: Donor

A 41-year-old male fell into a freshwater lake. Emergency responders pulled him from the lake and return of spontaneous circulation was achieved after twenty minutes of resuscitation. Subsequent chest radiographs showed development and worsening of bilateral opacities suspicious for pneumonia. Brain death was declared three days after hospital admission.

## 3. Case Presentation: Recipient

A 66-year-old male actively listed for a kidney and liver transplant presented to the surgical intensive care unit with sepsis and an oxacillin-sensitive* Staphylococcus aureus *bacteremia. He was treated with appropriate antibiotic therapy. He then developed acute tubular necrosis requiring continuous venovenous hemofiltration (CVVH). The patient had a chest X-ray showing mild pulmonary congestion and bronchoscopy was not performed as the patient did not show signs of pneumonia. After two weeks within his admission, the patient was treated for* Escherichia coli* bacteremia with piperacillin-tazobactam. Repeat blood cultures were negative after two days of therapy.

**Figure 1:**
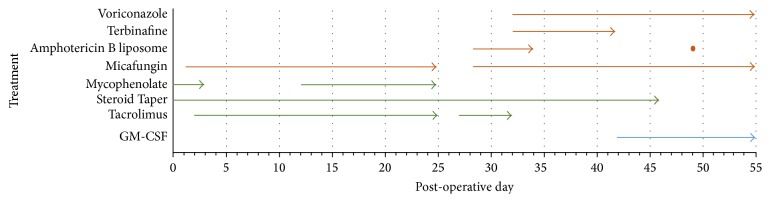
Antifungal agents and immunosuppression management.

While the patient was hospitalized, a donor became available. The donor kidney and liver were grossly normal. The patient underwent a combined kidney and liver transplant for chronic kidney failure with associated liver cirrhosis caused by the hepatitis C virus and alcoholism. The explanted liver was cirrhotic without any malignancy. At the time of transplantation, the patient's Model for End Stage Liver Disease (MELD) score was 40, having been decompensated in the surgical intensive care unit for the previous month. A summary of the patient's immunosuppression and antifungal management after transplant is provided in Figure 1.

The patient was given methylprednisolone 500 mg and mycophenolate mofetil 1,000 mg intraoperatively as induction immunosuppression. Maintenance immunosuppression protocols were followed after transplant. CVVH was performed intraoperatively and was discontinued on postoperative day (POD) 1 when renal graft function improved. The patient's hospital stay was complicated by a contained urine leak from the ureteroureterostomy which was managed nonoperatively with drains and ureteral stents. On POD 8, the patient was transferred out of the intensive care unit and on POD 14, to the inpatient rehab care unit as graft function continued to improve. The hepatic transaminases fluctuated and, on POD 25, he was transferred back to the surgical intensive care unit with peritonitis. As infection was suspected, immunosuppression was restricted to the use of corticosteroids tapered down to prednisone 5 mg daily. The patient was taken back to the operating room on POD 28 for a peritoneal washout. Cultures obtained during the procedure are presented in Tables 1 and 2. Of most concern was the* S. apiospermum *isolated from the perihepatic, perinephric, and biloma fluid. Endoscopic Retrograde Cholangiopancreatography (ERCP) was performed the following day, which did not reveal any extravasation. Discovery of* S. apiospermum* prompted contact with the Centers for Disease Control and Prevention (CDC), the Disease Transmission Advisory Committee (DTAC) of the United Network for Organ Sharing (UNOS), and the transplant centers treating recipients of allografts from the same donor. Other recipients had no evidence of infection.

**Figure 2:**
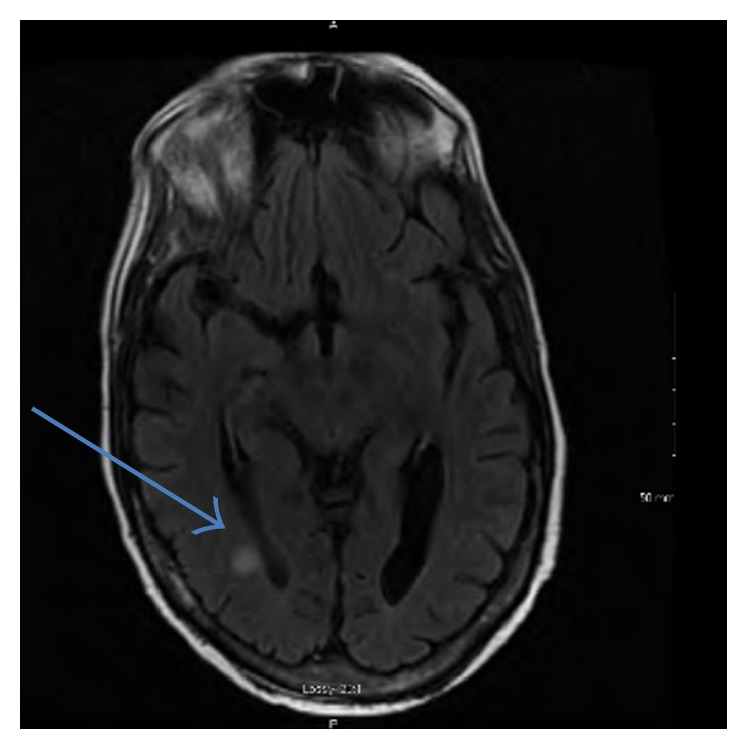
MRI of the brain. Multiple ring-enhancing lesions with associated diffusion restriction and T2/FLAIR hyperintensity are present throughout the supratentorial white matter.

On POD 28, due to worsening hemodynamics and peritonitis, another laparotomy and another washout were performed to evacuate an infected hematoma. The entire peritoneal surface was lined with mold. On POD 32, a mold suggestive of* Scedosporium *spp. grew from surgical cultures. Expert opinion for the management of our patient's suspected* S. apiospermum* infection was to use voriconazole targeting a trough of 2–4 mcg/mL, which we started on POD 32. Additionally, terbinafine was started for suspected synergy with voriconazole. A sample of the* S. apiospermum* was sent out for synergy studies which were expected to take a week or more to be conclusive. On POD 41* S. apiospermum* was identified. Laboratory results suggested terbinafine was not synergistic with voriconazole. Additionally, terbinafine is distributed rapidly to skin and bone and as such is not distributed well into visceral tissue and accumulation of terbinafine may cause hepatotoxicity. In response, terbinafine was discontinued and granulocyte macrophage colony-stimulating factor (GM-CSF) initiated on POD 42. The patient developed neurologic deficits with decreased vision to the right side and blurred vision bilaterally on POD 46. Magnetic resonance imaging (MRI) of the brain, shown in Figure 2, revealed multiple ring-enhancing lesions in the supratentorial compartment consistent with hematogenous central nervous system (CNS) scedosporiosis. We continued treatment with antifungal therapy. He developed septic shock and expired on POD 55.

## 4. Discussion

Differential diagnoses for opportunistic pathogens causing pneumonia related to near-drowning events include several key pathogenic bacteria and fungi. Gram negative bacteria are most often the causative agents in near-drowning cases [13].* Aeromonas spp.* have been described most often in near-drowning pneumonia cases.* Aeromonas *spp. naturally thrive in fresh and brackish waters and have been isolated in humans in wound and gastrointestinal infections after exposure to contaminated water. Other potential bacterial pathogens include* Pseudomonas aeruginosa*,* Legionella *spp.,* Klebsiella *spp., and other Enterobacteriaceae [13].* Scedosporium *spp. are the most common cause of invasive fungal infection, including pneumonia, CNS disease, and dissemination following near-drowning.

We identified 60 published cases of scedosporiosis after solid organ transplantation between the year 2000 and the present. Few were cases of scedosporiosis after solitary liver transplantation (5/60); most were reported following solitary lung (18/60) or kidney (18/60) transplantation.  Table 3 [2, 12, 14–42] provides a summary of the comprehensive literature review performed.* S. apiospermum *was isolated in the majority of cases, though* S. prolificans* and* S. aurantiacum* were also isolated. In four cases, both* S. apiospermum *and* S. prolificans* were identified in the same patient. In patients who had infections with a single isolate of* Scedosporium *spp., the observed mortality rates of* S. apiospermum, S. prolificans*, and* S. aurantiacum* were 54.5% (24/44), 70% (7/10), and 100% (3/3), respectively (excluding four cases with combined infections). Observed mortality of all* Scedosporium *spp. infections was 59% (36/61) including our patient case.

Kim et al. reported three fatal and two nonfatal cases of scedosporiosis following solid organ transplantation from the same donor who was victim to a near-drowning accident [12]. Additionally, they reviewed national Korean data on transplants and found that, among 2600 deceased-donor transplants over thirteen years, 27 (1%) of donors were victims of drowning. We accessed data from the United States Organ Procurement and Transplantation Network and found a similar rate: in 2015, 102/7586 (approximately 1.3%) of solid organ donors died of near-drowning events [43]. While this is a low percentage of all donors, the significance of our case is enhanced by the 58.3% mortality rate of infections involving* Scedosporium* spp. after solid organ transplantation.

At our center, liver transplant recipients are given antifungal prophylaxis based on their risk level. We stratify higher risk patients as those with MELD scores greater than 35, renal failure, requiring hemodialysis or CVVH prior to transplantation, recurrent spontaneous bacterial peritonitis, or preoperative prealbumin less than 10. High risk liver transplant recipients receive micafungin intravenously unless resistant* Candida glabrata*,* Aspergillus *spp., or* Cryptococcus *spp. are suspected; in such cases, amphotericin B lipid complex or voriconazole would be used. Our patient was classified as high risk and given micafungin prophylaxis.

All cases of suspected donor-derived infections should be reported to the DTAC. Communications with the CDC, DTAC, and the centers treating the recipients of the other organs provided by this donor revealed the recipient of the heart was receiving voriconazole prophylaxis, while the recipients of the other kidney and the pancreas were not. To the best of our knowledge, no other recipient of this donor's organs is infected with* Scedosporium *spp. The other centers were advised by the CDC to commence voriconazole prophylaxis for an undetermined duration. Expert opinion for the management of our patient's scedosporiosis was voriconazole targeting a trough of 2–4 mcg/mL. Additionally, immunosuppression was to be limited or discontinued to improve infection clearance.

*S. apiospermum* is inherently resistant to amphotericin B, including the lipid formulations. Of the newer triazole antifungals, data on the* in vitro* activity of voriconazole is most robust, showing activity against* S. apiospermum* with MICs of 0.12 to 0.5 mcg/mL in clinical isolates [30]. Because of poor activity with single agents, various antifungal combinations have been examined for efficacy against* S. apiospermum*. Strong synergy was found* in vitro* between voriconazole and terbinafine against clinical isolates of* Scedosporium *spp. [30]. Combination of micafungin and voriconazole has demonstrated a synergistic effect against several fungi* in vitro* including* Scedosporium *spp. The synergistic mechanism may include reorganization of the cell wall allowing increased exposure of beta-glucan to the immune system [44].

In addition to antifungal agents, GM-CSF has been studied with some success [44, 45]. While antifungal therapy remains crucial to recovery, the treatment of scedosporiosis infections depends on the function of the host's innate immune system, in particular, polymorphonuclear cells (PMNs). The mechanism of GM-CSF increases the antifungal action of PMNs* in vitro* [45].

Despite good allograft function, our patient did not survive disseminated infection with* S. apiospermum* after combined kidney and liver transplantation. The exact mode of transmission and acquisition of* S. apiospermum *in this patient remains uncertain. We suspect donor to recipient transmission as the donor was the victim of a near-drowning event and had chest radiographs suspicious for pneumonia; however, we cannot rule out recipient colonization or nosocomial infection after the transplant.

Infections caused by* Scedosporium *spp. following solid organ transplant, while not common, are often fatal for recipients. There are no standards of practice for prophylaxis for patients at risk for developing scedosporiosis, such as recipients of organs from nearly drowned donors. Considering our case and the scedosporiosis mortality rate over 50% and the low rate of nearly drowned donors, we recommend screening for* Scedosporium *spp. when donated organs originate from a near-drowning victim.

## Figures and Tables

**Table 1:** Culture results.

Date	Culture site	Source	Result
POD 28	Abdominal fluid	Surgical specimen	*C. tropicalis*
			*E. faecalis*
			*E. coli*
			*M. morganii*

POD 28	Biloma fluid	Surgical specimen	*E. faecium*
			*E. faecalis*
			*S. apiospermum*
			*C. tropicalis*

POD 28	Perihepatic fluid	Surgical specimen	*S. apiospermum*
			*E. faecium*
			*C. tropicalis*

POD 28	Perinephric hematoma tissue	Surgical specimen	*S. apiospermum*

POD 32	Abdominal clot	Surgical specimen	*S. apiospermum*

POD 32	Intestinal serosal tissue	Surgical specimen	*S. apiospermum*
			*E. faecium*

POD 50	LUQ abdominal fluid	Surgical specimen	*C. glabrata*
			*C. tropicalis*

POD 50	Cerebrospinal fluid	Lumbar puncture	Negative

**Table 2:** Susceptibility testing of fungal isolates.

Isolate	Resistant (MIC)	Intermediate (MIC)	Susceptible (MIC)
*Scedosporium apiospermum*	5-Fluorocytosine (>64), amphotericin B (>16), caspofungin (>8), micafungin (>8), terbinafine (>2)	None	Voriconazole (1)

*Candida tropicalis*	None	None	Caspofungin (≤0.25), fluconazole (≤1), voriconazole (≤0.12)

*Candida glabrata*	None	None	Caspofungin (2), fluconazole (≤1), voriconazole (≤0.12)

**Table 3:** Review of solid organ transplant-associated *Scedosporium* infection.

Ref #	Year	Recipient information		Recipient organ(s)	Species of *Scedosporium*	Treatment approach	Outcome
		Age (years)	Gender				
[14]	2000	67	Male	Heart	*apiospermum*	Voriconazole	Deceased

[15]	2001	42	Female	Heart/lung	*apiospermum*	Fluconazole, itraconazole	Deceased
		22	Female	Heart/lung	*prolificans *+* apiospermum*	Fluconazole, itraconazole	Deceased
		42	Male	Heart/lung	*prolificans + apiospermum *	Fluconazole, itraconazole	Alive
		49	Male	Lung	*prolificans + apiospermum*	Fluconazole, itraconazole	Deceased
		39	Male	Lung	*prolificans + apiospermum*	Fluconazole, itraconazole	Alive
		52	Female	Lung	*apiospermum*	Fluconazole, itraconazole	Alive
		38	Female	Heart/lung	*apiospermum*	Fluconazole, itraconazole	Deceased

[16]	2002	58	Female	Liver	*apiospermum*	Itraconazole, miconazole	Alive
		37	Male	Lung	*apiospermum*	Itraconazole	Deceased
		30	Male	Lung	*apiospermum*	Amphotericin B, miconazole	Deceased
		37	Female	Heart/lung	*apiospermum*	Miconazole	Deceased
		39	Male	Liver	*apiospermum*	5-Flucytosine, amphotericin B	Deceased
		67	Male	Heart	*apiospermum*	Itraconazole, voriconazole	Deceased
		36	Male	Kidney	*apiospermum*	Itraconazole, miconazole	Deceased

[17]	2002	49	Male	Kidney	*apiospermum*	Voriconazole	Deceased

[18]	2002	62	Female	Kidney	*apiospermum*	None	Deceased
		58	Male	Kidney	*apiospermum*	Itraconazole, voriconazole	Alive

[19]	2002	64	Female	Lung	*apiospermum*	Amphotericin B, itraconazole	Deceased

[20]	2002	71	Male	Heart	*apiospermum*	Itraconazole	Alive

[21]	2003	24	Male	Lung	*apiospermum*	Voriconazole	Alive
		59	Male	Kidney	*apiospermum*	Voriconazole	Alive

[22]	2004	50	Male	Kidney	*apiospermum*	Voriconazole	Alive

[23]	2004	58	Male	Kidney	*apiospermum*	Amphotericin B, fluconazole, itraconazole, miconazole	Alive

[24]	2004	56	Female	Lung	*prolificans*	Voriconazole	Deceased

[25]	2005	55	Male	Small bowel	*prolificans*	Amphotericin B	Deceased
		40	Male	Kidney/pancreas	*prolificans*	Voriconazole	Alive
		67	Male	Kidney	*apiospermum*	Amphotericin B	Alive
		51	Female	Small bowel	*prolificans *	Amphotericin B, Voriconazole, caspofungin	Deceased
		67	Male	Heart	*apiospermum *	Voriconazole	Deceased
		17	Male	Liver	*prolificans *	Voriconazole	Deceased
		64	Male	Liver	*apiospermum*	None	Deceased
		45	Male	Heart	*apiospermum*	Itraconazole	Deceased
		56	Male	Liver	*apiospermum *	Voriconazole	Deceased
		44	Female	Heart	*prolificans *	Amphotericin B	Deceased
		68	Male	Kidney	*prolificans *	Voriconazole	Alive
		52	Male	Small bowel	*apiospermum *	Amphotericin B, Voriconazole, caspofungin	Alive
		62	Male	Kidney/pancreas	*apiospermum *	Voriconazole	Alive

[26]	2005	26	Female	Lung	*apiospermum*	Miconazole, voriconazole	Deceased

[27]	2006	58	Male	Kidney	*apiospermum*	Miconazole, voriconazole	Alive

[28]	2006	57	Male	Lung	*apiospermum*	Terbinafine, voriconazole	Alive
		63	Male	Lung	*apiospermum*	Liposomal amphotericin B, terbinafine, voriconazole	Alive

[29]	2007	59	Female	Kidney	*apiospermum*	Voriconazole	Alive

[30]	2007	43	Male	Lung	*apiospermum*	Caspofungin, itraconazole, liposomal amphotericin B	Deceased

[31]	2008	70	Male	Kidney	*prolificans*	Terbinafine, voriconazole	Alive

[32]	2009	65	Female	Kidney	*apiospermum*	Voriconazole	Alive

[33]	2010	37	Female	Lung	*apiospermum*	Caspofungin, terbinafine, voriconazole	Deceased

[34]	2011	16	Female	Lung	*apiospermum*	Voriconazole	Alive

[35]	2012	35	Male	Lung/liver	*apiospermum*	Caspofungin, voriconazole	Deceased

[36]	2012	37	Female	Lung	*apiospermum*	Aerosolized amphotericin B, caspofungin, itraconazole, voriconazole	Deceased

[37]	2013	17	Female	Lung	*apiospermum*	Caspofungin, posaconazole, voriconazole	Alive

[38]	2013	70	Female	Lung	*prolificans*	Caspofungin, terbinafine, voriconazole	Deceased

[39]	2014	50	Female	Kidney	*apiospermum*	Voriconazole	Alive

[40]	2014	35	Male	Kidney	*prolificans*	Itraconazole, liposomal amphotericin B, micafungin, voriconazole	Deceased

[41]	2015	70	Male	Heart	*apiospermum*	Posaconazole, terbinafine	Deceased

[12]	2015	19	Male	Heart	*aurantiacum *	Amphotericin B prophylaxis	Deceased
		56	Male	Kidney	*aurantiacum *	Itraconazole, liposomal amphotericin B	Deceased
		57	Female	Kidney	*aurantiacum*	Caspofungin, voriconazole	Deceased

[2]	2015	40	Male	Kidney	*apiospermum*	Voriconazole	Deceased

[42]	2015	18	Female	Lung	*apiospermum*	Terbinafine, voriconazole	Alive
